# Tea Polyphenols Enhanced the Antioxidant Capacity and Induced Hsps to Relieve Heat Stress Injury

**DOI:** 10.1155/2021/9615429

**Published:** 2021-08-06

**Authors:** Bin Yin, Ruirui Lian, Zhen Li, Yueyue Liu, Shifa Yang, Zhongli Huang, Zengcheng Zhao, Ying Li, Chuanxi Sun, Shuqian Lin, Renzhong Wan, Guiming Li

**Affiliations:** ^1^Poultry Institute, Shandong Academy of Agricultural Science, Jinan, Shandong 250023, China; ^2^Shandong Provincial Animal and Poultry Green Health Products Creation Engineering Laboratory, Shandong Academy of Agricultural Science, Jinan, Shandong 250023, China; ^3^Laboratory of Pharmacology and Toxicology Research, College of Animal Science and Veterinary Medicine, Shandong Agricultural University, Taian 271000, China

## Abstract

Keap1-Nrf2-ARE and heat shock proteins (Hsps) are important endogenous protection mechanisms initiated by heat stress to play a double protective role for cell adaptation and survival. H9C2 cells and 80 300-day-old specific pathogen-free chickens were randomly divided into the control and tea polyphenol groups and used to establish a heat stress model in vitro and in vivo. This task was conducted to explore the protection and mechanism of tea polyphenols in relieving thermal injury. A supplement with 10 *μ*g/mL tea polyphenols could effectively relieve the heat damage of H9C2 cells at 42°C. Accordingly, weaker granular degeneration, vacuolar degeneration, and nucleus deep staining were shown. A strong antioxidant capacity was manifested in the upregulation of the total antioxidant capacity (T-AOC) (at 5 h, *P* < 0.05), *Hemeoxygenase-1 mRNA* (at 2 h, *P* < 0.01), superoxide dismutase (SOD) (at 2, 3, and 5 h, *P* < 0.05), and Nrf2 (at 0 and 5 h, *P* < 0.01). A high expression of Hsps was reflected in CRYAB at 3 h; Hsp27 at 0, 2, and 3 h (*P* < 0.01); and Hsp70 at 3 and 5 h (*P* < 0.01). The supplement with 0.2 g/L tea polyphenols in the drinking water also had a good effect in alleviating the heat stress damage of the myocardial cells of hens at 38°C. Accordingly, light pathological lesions and downregulation of the myocardial injury-related indicators (LDH, CK, CK-MB, and TNF-*α*) were shown. The mechanism was related to the upregulation of T-AOC (at 0 h, *P* < 0.05), GSH-PX (at 0.5 d, *P* < 0.01), SOD (at 0.5 d), and Nrf2 (at 0 d with *P* < 0.01 and 2 d with *P* < 0.05) and the induced expression of CRYAB (at 0.5 and 2 d), Hsp27 (at 0, 0.5, and 5 d), and Hsp70 (at 0 and 0.5 d). In conclusion, the tea polyphenols enhanced the antioxidant capacity and induced Hsps to relieve heat stress injury.

## 1. Introduction

Heat stress refers to a series of nonspecific reactions caused by high temperature exceeding the upper limit of the isothermal zone, especially in the tropical areas and in summer. Poultry has the characteristics of rich feathers, no sweat glands, strong metabolism, and high body temperature. Accordingly, the production performance of poultry is easily affected by ambient temperature [1, 2]. Studies have shown that the laying hen's appetite and body temperature regulation ability were reduced when the house temperature was greater than 30°C, thereby resulting in a significant decrease in the weekly egg production rate and weight [3]. When the house temperature reached 34°C, the weekly mortality was greatly increased. Myocardial cells are sensitive to heat. The H9C2 cells exhibited granular degeneration after 1 h, vacuolar degeneration after 2 h, and karyopyknosis after 5 h when they were exposed to heat stress [4]. The damage of such cells is an important factor that leads to sudden death. The reason for the injury was related to the imbalance of redox and protein homeostasis, which were caused by the production of excessive reactive oxygen species (ROS) and by the denaturation and aggregation of proteins, respectively [5]. Screening antioxidant additives is an effective method to minimize heat injury. In our previous studies, feeding broiler chicken natural substances, such as coenzyme Q10 and vitamin C, during heat stress showed a weaker injury of myocardial cells than the heat-only group [4, 6]. Therefore, antioxidant additives have been given attention to alleviate heat stress.

The main field of application of natural products is in the prevention of oxidation of animals and their products [7, 8]. Tea as a natural additive has health benefits because of its biological properties [9]. Tea polyphenols were extracted from tea leaves, rich in hydroxyl, and contain the same main structure 2-phenylbenzopyran, which was the foundation of an antioxidant activity [10]. Tea polyphenols, a new type of green feed additive, were used in animal farming, and their biological activity had attracted widespread attention [11]. The supplement of epigallocatechin-3-gallate could increase the activity of antioxidant enzymes, such as SOD and GSH, to broiler chickens under heat stress and to hens to improve the antioxidant activity of eggs [12]. In addition, 50 *μ*M EGCG incubated on BME-UV1 cells for 48 h could prevent H_2_O_2_-induced oxidative lipid damage by a higher concentration of intracellular GSH; EGCG is suggested as a topical treatment for the control of bovine intramammary inflammation [13]. A meta-analysis found that the addition of tea polyphenols in the diet could significantly increase the egg production rate, average egg weight, and color of the yolk and could reduce the feed-to-egg ratio [14]. However, the mechanism is obscured.

Keap1-Nrf2-ARE and HSF1-HSE are important endogenous protection mechanisms that regulate the expression of cellular antioxidant enzymes and heat shock proteins (Hsps) and play a double protective role for cell adaptation and survival under heat stress, respectively [5, 15]. *Petasites japonicus* could alleviate UVB-induced apoptosis via HSF1-activated Hsps and Nrf2-activated HO-1 [16]. Solanesol could protect the human liver cells from ethanol-induced oxidative damage via the upregulation of HO-1 and Hsp70 [17]. In the Keap1-Nrf2-ARE pathway, Nrf2 normally binds to Keap1 and isolates in the cytoplasm as a stable complex; the proteasome degradation mechanism was disrupted when simulated. The novosynthetized Nrf2 was free to transfer to the nucleus and combined with the antioxidant response element (ARE) to drive the expression of antioxidant genes, such as NQO1, HO-1, SOD, and GSH-PX [5, 18]. The HSF1-HSE pathway was a defensive adaptive response characterized by the activation of Hsps, which as a molecular chaperone, could help protein correctly folding, translocation, renaturation, and degradation to effectively alleviate the imbalance of protein homeostasis [5, 18]. Drug stimulation, such as rosemary and coenzyme Q10, could increase the survival rate of cells under heat stress conditions by upregulation of Hsp70 [6, 19, 20]. Meanwhile, the protection decreased when quercetin, an Hsp70 inhibitor, was used [21]. The overexpression of CRYAB could significantly improve the heat resistance of H9C2 by stabilizing the cytoskeletal structure and regulating the cell cycle. The protection was positively correlated with the amount of CRYAB overexpression [22].

We hypothesized that tea polyphenols could enhance cardiomyocyte heat resistance by improving the antioxidant capacity and heat shock response. We established in vivo and in vitro heat stress models to investigate the hypothesis. Thereafter, different durations of heat stress are applied to clarify the role of tea polyphenols in cardiomyocyte protection during various periods of heat stress and in the activation effects of antioxidation indicators and Hsps.

## 2. Materials and Methods

### 2.1. Cell Culture

H9C2 cells, derived from embryonic rat hearts, were purchased from the American Type Culture Collection. The cells were seeded in plastic tissue culture flasks (Corning, China) at a density of 1 × 10^5^/mL and cultured in Dulbecco's Modified Eagle Medium (Gibco, USA) supplemented with 10% fetal bovine serum (Biological Industries, BI), 100 U penicillin, and 100 *μ*g/mL streptomycin (Gibco). Thereafter, the cells were incubated in a 5% CO_2_ humidified atmosphere (Thermo, USA) at 37°C. When the cells formed a complete monolayer, they were passaged within 30 passages for the subsequent experiments.

### 2.2. Screening the Optimal Concentration of Tea Polyphenols

Concentration screening was performed according to the cell viability measured by cell counting kit-8 (CCK-8, Beyotime, China). The cells were seeded in 96-well plates. When the cell fusion degree reached 70%–80%, the medium was replaced with a fresh one containing tea polyphenols at 0, 5, 10, 20, 40, 60, or 80 *μ*g/mL. The tea polyphenols was purchased from Jiangsu Dehe Biological Technology Co., Ltd., with polyphenol content > 98%, catechins > 80%, and epigallocatechin gallate (EGCG) > 59%. The plate was incubated at 37°C for 16 h, and the medium was replaced with a fresh medium containing 10% CCK-8 for incubation for 2 h at 37°C. Absorbance was measured at 450 nm with a microplate reader (NanoQuant, USA) to obtain the maximum safe concentration of tea polyphenols. After the cells were incubated with different concentrations of tea polyphenols for 16 h, they were transferred to a 42°C 5% CO_2_ cell incubator for a 5 h heat stress to discriminate the protection of tea polyphenols under heat stress. The medium was replaced with a fresh one containing 10% CCK-8 for incubation for 2 h at 37°C, and the absorbance was detected. GraphPad Prism 6.01 was used for data analysis.

### 2.3. In Vitro Myocardial Cell Heat Stress Model

**Figure 1:**
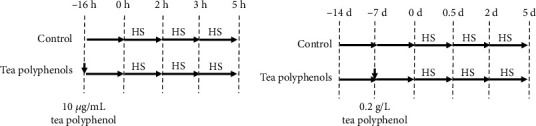
Heat stress model establishment. (a) Heat stress model in vitro. The H9C2 cells were divided into control and tea polyphenol groups. When the cells grew to a fusion degree of 70–80%, the tea polyphenol group was treated with 10 *μ*g/mL tea polyphenols for 16 h and then suffered heat stress at 42°C for 0, 2, 3, and 5 h. (b) Heat stress model in vivo. Eighty 300-day-old specific pathogen-free chickens were divided into control and tea polyphenol groups. The chickens in the tea polyphenol group were supplied with 0.2 g/L tea polyphenols in drinking water for 7 d. Then, heat stress was triggered at 38°C for 0, 0.5, 2, and 5 d.

The cells were randomly divided into the control and tea polyphenol groups to evaluate the heat stress protection of tea polyphenols. When the cells grew to a fusion degree of 70–80%, the control group medium was changed to a fresh one. Meanwhile, the tea polyphenol group medium was changed to a fresh one containing 10 *μ*g/mL tea polyphenols. After a continued culturing for 16 h, the cells were transferred into a 42°C 5% CO_2_ cell incubator for 0, 2, 3, and 5 h heat stress (Figure 1(a)). Thereafter, the cells and supernatants were immediately harvested for analysis.

### 2.4. Histological Analysis of the H9C2 Cells

The H9C2 cells were seeded on coverslips and treated as the vitro heat stress model. The cells were collected, fixed in 4% paraformaldehyde for 30 min, and stained with haematoxylin for 5 min and with eosin for 3 min. Then, the cells were immediately passed through the gradient ethanol consisting of 75, 85, 95, 95, 100, and 100% for 2 min. After the cells were passed through xylene for 2 min, the coverslips were mounted using neutral resin and analyzed via light microscopy with an Axio Imager.A2 instrument (Zeiss, German).

### 2.5. Oxidative Damage-Related Indicator Analysis of H9C2

The H9C2 cells, seeded in 30 mm dishes, were treated as the vitro heat stress model, and the cells were collected for the detection of antioxidant indicators *Hemeoxygenase-1* (*HO-1*) *mRNA*, total antioxidant capacity (T-AOC), and superoxide dismutase (SOD). T-AOC was performed with commercial kits in accordance with the manufacturer's instructions (A012-5, Nanjing Jiancheng). SOD was measured by Kingmed Diagnostics. The *HO-1 mRNA* level was analyzed by RT-PCR. The total RNA was extracted using an RNAiso Plus reagent (TaKaRa, Japan) and quantified with a NanoDrop 2000 (Thermo, USA) by the absorbance at 260 nm and A260/A280 ratio. Reverse transcription was then carried out with a real-time quantitative PCR (RT-PCR kit) (Vazyme, China) to synthesize the cDNA, which was used for RT-PCR with a Power SYBR Green Master Mix (Vazyme) according to the manufacturer's instructions. The relative transcription level of *HO-1* was normalized against glyceraldehyde-3-phosphate dehydrogenase (GAPDH) and quantified using the comparative Ct (2^−*ΔΔ*Ct^) method. The primers for HO-1 were forward: 5′-CTTTCAGAAGGGTCAGGTGTC-3′, and reverse: 5′-TGCTTGTTTCGCTCTATCTCC-3′. Meanwhile, the primers for GAPDH were forward: 5′-GCAAGTTCAACGGCACAG-3′, and reverse: 5′-GCCAGTAGACTCCACGACAT-3′.

### 2.6. Dynamic Expression Levels of Nrf2, Hsps, and Cleaved-Caspase 3 in the H9C2 Cells

The dynamic expression levels were analyzed using western blot. The treated cells were washed with PBS three times and lysed with a RIPA lysis buffer (R0010, Solarbio) containing a 1% protease inhibitor (Nanjing Jiancheng Biochemical Reagent) in 4°C for 15 min. The supernatants were collected after centrifugation at 12,000 g for 10 min, and the protein concentration was measured with a BCA Protein Assay Kit (P0009; Beyotime). A 5x sodium dodecyl sulfate polyacrylamide gel electrophoresis (SDS-PAGE) loading buffer was added to the protein samples. The samples were then boiled for 15 min and stored at −20°C until needed. Approximately 15 *μ*g of each protein sample was separated by 10% SDS-PAGE and transferred onto polyvinylidene fluoride membranes. After the membranes were blocked with 5% nonfat dry milk in tris-buffered saline and Tween 20 (TBST) buffer for 2 h, they were incubated with anti-Nrf2 (1 : 1000, Abcam), anti-Hsp70 (1 : 1000, Enzo), anti-Hsp27 (1 : 1000, Santa), anti-CRYAB (1 : 1000, CST), and anticleaved-caspase 3 (1 : 500, CST) at 4°C overnight. The membranes were washed with TBST and then incubated with anti-mouse IgG HRP conjugated (1 : 3000, CST) or anti-rabbit IgG HRP conjugated (1 : 3000, CST) secondary antibodies at room temperature for 2 h. After the membranes were washed with TBST, they were added with an enhanced chemiluminescence detection regent ECL (Thermo, USA) and detected by the ImageQuant LAS 500 digital imaging system (GE Healthcare, Japan). The intensity of the scanned bands was determined using Quantity One.

### 2.7. In Vivo Myocardial Cell Heat Stress Model

Eighty 300-day-old specific pathogen-free chickens were purchased from Shandong Health-Tec Laboratory Animal Breeding Co., Ltd., Jinan, China. The chickens were randomly divided into control and tea polyphenol groups. The experimental design is shown in Figure 1(b). All chickens were fed for adaptive growth for 7 d in the normal environment (23 ± 2°C, approximately 60% humidity) with free diet and drinking. Followed by a 7 d dosing period, the tea polyphenol group was fed with water supplemented with 0.2 g/L tea polyphenols. Meanwhile, the control group was fed with normal water. Then, heat stress (37 ± 1°C) was triggered for 0, 0.5, 2, and 5 d with the same drinking with dosing period. After the heat stress, the serum and heart tissues were immediately collected for analysis. All experiments were performed in accordance with the guidelines of the Animal Ethics Committee of Shandong Province (China) and approved by the Institutional Animal Care and Use Committee of Shandong Academy of Agricultural Sciences (SAAS-2019-032), China.

### 2.8. Pathological Examination of the Myocardial Tissues

The myocardial tissues fixed in 10% formalin were resized to 5 mm × 5 mm × 2 mm and then refixed in 10% formalin for 12 h. After 75%, 85%, 95%, 95%, 100%, and 100% ethanol gradient dehydration of each gradient for 1 h, the myocardial tissues were immersed in xylene for 20 min for transparency. Transparent tissues were embedded in paraffin wax and sliced into 5 *μ*m thick serial sections. The sections were deparaffinized in xylene, rehydrated with reduced alcohol series, and stained with haematoxylin for 5 min and eosin for 2 min. The slices were sealed with coverslips using a neutral resin for light microscopic analysis with an Axio Imager.A2 instrument (Zeiss, German).

### 2.9. Injury and Oxidation-Related Index Analysis in the Chicken Serum

The lactate dehydrogenase (LDH), creatine kinase (CK), CK isoenzyme MB (CK-MB), and SOD in the chicken serum were sent to Kingmed Diagnostics for detection. The levels of TNF-*α* (JYM0033Ch, Wuhan Colorful Gene Biotechnology Co., Ltd.), malondialdehyde (MDA, A003-1-2), total antioxidant capacity (T-AOC, A012-5), and glutathione peroxidase (GSH-PX, A005-1-2) in the chicken serum were determined with kits in accordance with the manufacturer's instructions purchased from Nanjing Jiancheng Bioengineering Institute.

### 2.10. Dynamic Expression Levels of Nrf2, Hsps, and Cleaved-Caspase 3 in the Myocardial Tissues

Approximately 20 mg of myocardial tissue for each chicken was homogenized in a 200 *μ*L RIPA buffer (R0010, Solarbio) containing 1% PMSF and lysed at 4°C for 30 min. The supernatant was collected after centrifugation at 12,000 g for 10 min. The protein concentration was measured with a BCA Protein Assay Kit (P0009; Beyotime). The 5x SDS-PAGE loading buffer was added to the protein samples. The samples were then boiled for 15 min and stored at −20°C until needed. Approximately 20 *μ*g of each protein sample was used for western blot analysis as the method in H9C2 cells.

### 2.11. Statistical Analysis

Data differences between the experimental groups were analyzed by SPSS software v.20.0 by one-way analysis of variance and least significant difference multiple comparison test methods. Statistically significant differences were set at *P* < 0.05 (^∗^ or ^#^). Extremely significant differences were set at *P* < 0.01 (^∗∗^ or ^##^).

## 3. Results

### 3.1. Optimal Concentration of Tea Polyphenols

**Figure 2:**
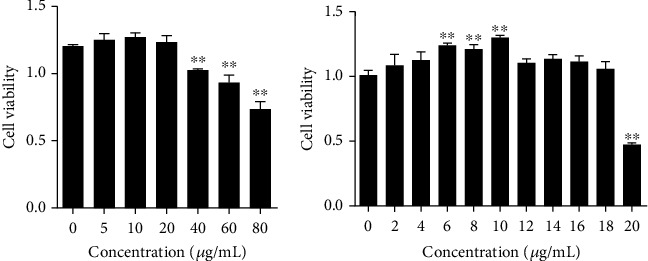
Viability of H9C2 cells. (a) Cell viability upon addition of various concentrations of tea polyphenols without heat stress. The addition of 20 *μ*g/mL of tea polyphenols had no effect on the H9C2 cells. However, 40 *μ*g/mL of tea polyphenols reduced the cell viability. (b) Cell viability upon addition of various concentrations of tea polyphenols following heat stress for 5 h. Approximately 10 *μ*g/mL of tea polyphenols presented a good protection.

The optimal concentration screening was performed according to the cell viability. Under a normal condition, the concentration of tea polyphenols in the range of 0–20 *μ*g/mL was safe for the H9C2 cells. However, a dose of 40 *μ*g/mL or higher could significantly lower the cell viability (*P* < 0.01); the cell viability decreased by 38.86% when the concentration reached 80 *μ*g/mL (Figure 2(a)). Under the condition of heat stress for 5 h, the protection of tea polyphenols first increased and then decreased with the increasing concentration; the 10 *μ*g/mL concentration presented the best, which could increase cell viability by 28.52% (Figure 2(b)). In summary, 10 *μ*g/mL of tea polyphenols was the optimal concentration and used in the subsequent protection experiments.

### 3.2. Histological Analysis of the H9C2 Cells

**Figure 3:**
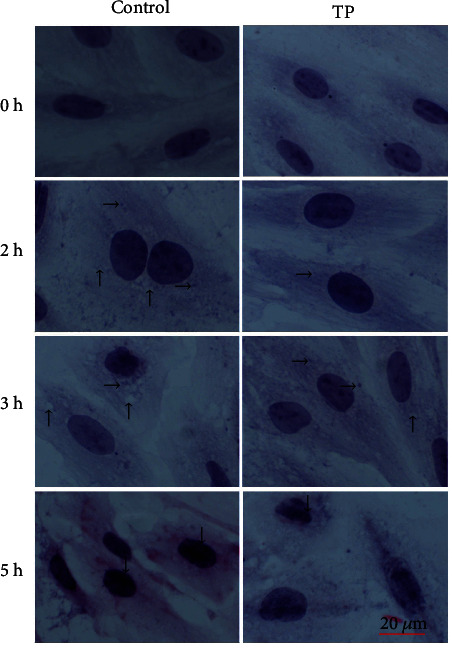
Pathological lesions caused by heat stress to H9C2 cells. TP means tea polyphenols. H9C2 myocardial cells, HE staining, 1 bar = 20 *μ*m. Granular degeneration (→), vacuolar degeneration (↑), and karyopyknosis (↓) occurred after heat stress. Approximately 10 *μ*g/mL tea polyphenols could, respectively, significantly weaken granular degeneration, vacuolar degeneration, and karyopyknosis at 2, 3, and 5 h.

Pathological lesions occurred when the H9C2 cells were exposed to heat stress (Figure 3). In the control group, the H9C2 cells demonstrated granular degeneration when they were exposed to heat stress for 2 h. Meanwhile, the supplement with 10 *μ*g/mL tea polyphenols could significantly weaken granular degeneration. When the heat stress continued for 3 h, the granular degeneration in the control group still existed, and large vacuolar degeneration was observed. By contrast, the cells in the tea polyphenol group presented granular degeneration and relatively small vacuolar degeneration. When the heat stress lasted for 5 h, nucleus deep staining was observed in the control group, while the tea polyphenol group was relatively light.

### 3.3. Oxidative Damage-Related Indicator Analysis of H9C2

**Figure 4:**
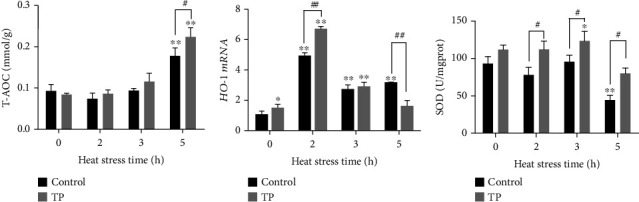
Indicators of oxidative damage after heat stress. TP means tea polyphenols. Heat stress increased the T-AOC (a) and *HO-1 mRNA* (b) but decreased the SOD (c) after heat stress. The supplement with 10 *μ*g/mL tea polyphenols showed a strong antioxidant capacity manifested in the upregulation of T-AOC (at 5 h, *P* < 0.05), *HO-1 mRNA* (at 2 h, *P* < 0.01), and SOD (at 2, 3, and 5 h, *P* < 0.05).

The levels of oxidative damage-related indicators T-AOC, *HO-1 mRNA*, and SOD in the H9C2 cells are shown in Figure 4. The heat stress could upregulate the T-AOC levels, which showed a significant difference at 5 h (*P* < 0.01). Such stress could upregulate the *HO-1 mRNA* levels and showed significant differences at 2, 3, and 5 h (*P* < 0.01). However, the level of SOD presented a downward trend and a significant difference at 5 h (*P* < 0.01). The supplement with 10 *μ*g/mL tea polyphenols could increase the total antioxidant capacity (T-AOC) of the H9C2 cells, and a significant difference was observed at 5 h (*P* < 0.05). Heat stress could upregulate the *HO-1 mRNA* levels and showed a significant difference at 2 h (*P* < 0.01). Moreover, heat stress could enhance the activity of the SOD level and presented differences at 2, 3, and 5 h (*P* < 0.05).

### 3.4. Dynamic Expression Levels of Nrf2, Hsps, and Cleaved-Caspase 3 in the H9C2 Cells

**Figure 5:**
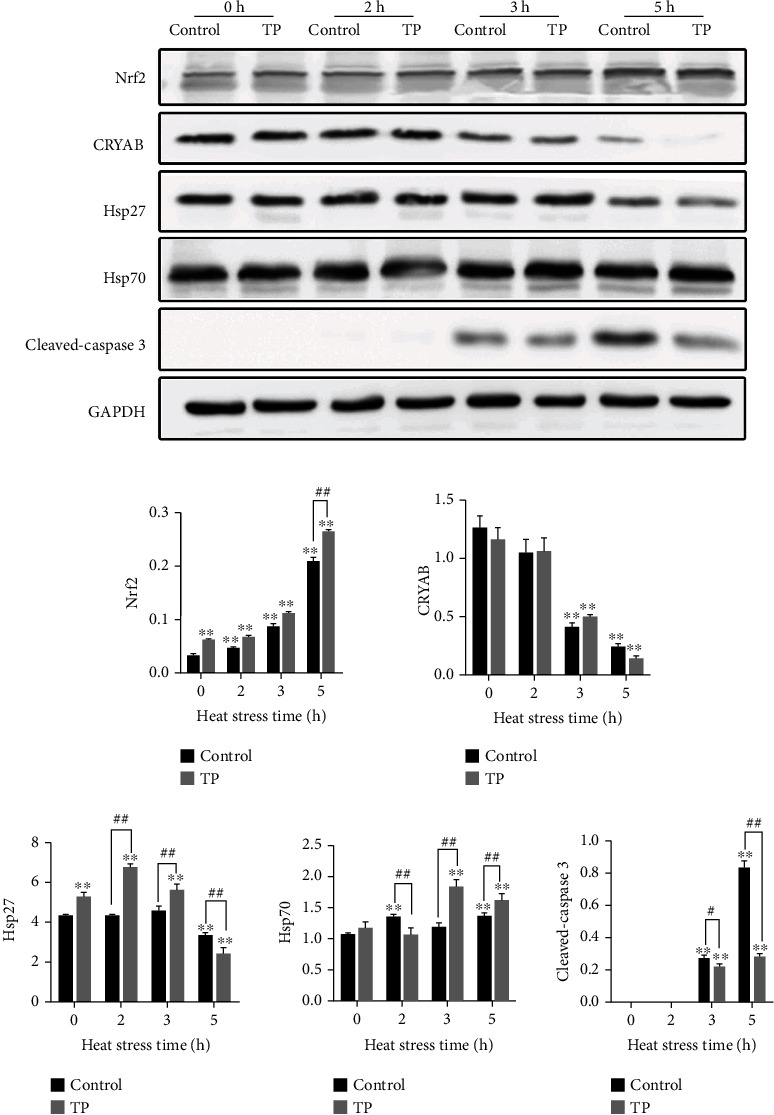
Dynamic expression levels for Nrf2, Hsps, and cleaved-caspase 3 in the H9C2 cells. TP means tea polyphenols. The expression levels were detected using western blot relative to the housekeeping protein GAPDH. The heat stress increased Nrf2, Hsp70, and cleaved-caspase 3 but decreased the level of CRYAB. The supplement with 10 *μ*g/mL tea polyphenols had high expressions of Nrf2 (at 0 and 5 h, *P* < 0.01) and Hsps reflected in CRYAB (at 3 h), Hsp27 (at 0, 2, and 3 h, *P* < 0.01), and Hsp70 (at 3 and 5 h, *P* < 0.01) but low caspase 3 (at 3 h with *P* < 0.05 and 5 h with *P* < 0.01). ^∗^*P* < 0.05 and ^∗∗^*P* < 0.01 were compared with 0 h in the control group. #*P* < 0.05 and *^##^P* < 0.01 were compared between the control and tea polyphenol groups under the same conditions.

The dynamic expression levels of Nrf2, Hsps, and cleaved-caspase 3 in the H9C2 cells are shown in Figure 5. Heat stress could increase the expression level of Nrf2 in the H9C2 cells, which showed a significant upregulation at 2, 3, and 5 h (*P* < 0.01). The supplement with 10 *μ*g/mL tea polyphenols could induce the expression of Nrf2 under normal condition (0 h of heat stress) and at 5 h of heat stress (*P* < 0.01). Heat stress could downregulate the level of CRYAB and showed a significant decrease at 3 and 5 h (*P* < 0.01). The initial stage of heat stress for Hsp27 had a slight effect on its expression until at 5 h of heat stress which showed a significant decrease (*P* < 0.01). For Hsp70, heat stress induced extremely significant increases at 2 and 3 h (*P* < 0.01). The supplement with 10 *μ*g/mL tea polyphenols increased the expression of CRYAB at 3 h of heat stress and could induce the expression of Hsp27 at 0, 2, and 3 h (*P* < 0.01) and Hsp70 at 3 and 5 h (*P* < 0.01). Heat stress increased the expression of cleaved-caspase 3, and a supplement with 10 *μ*g/mL tea polyphenols had a very significant relief effect at 3 and 5 h (*P* < 0.01).

### 3.5. Pathological Examination of Myocardial Tissue

**Figure 6:**
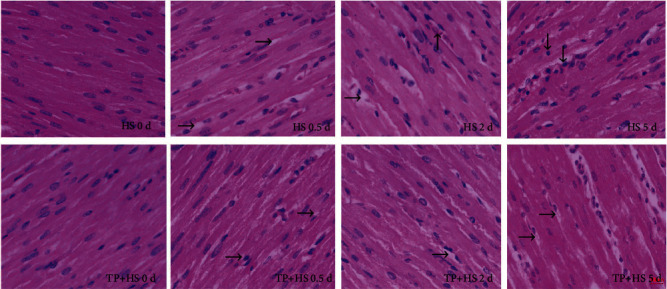
Pathological lesions caused by heat stress to laying cardiomyocytes. TP means tea polyphenols and HS means heat stress. Laying cardiomyocytes, HE staining, 1 bar = 20 *μ*m. Vacuolar degeneration (→), capillary vasodilation (↑), and cardiomyocyte necrosis (↓) occurred when the laying cardiomyocytes suffered from heat stress for 0.5, 2, and 5 d. The supplement with 0.2 g/L tea polyphenols in drinking water effectively alleviated heat stress-induced vacuolar degeneration and capillary vasodilation at 2 d and cardiomyocyte necrosis at 5 d.

The pathological changes of the laying hen myocardial tissue are shown in Figure 6. Heat stress damaged the myocardial tissue. Such damage became severe with the prolongation of heat stress. The cardiomyocytes demonstrated vacuolar degeneration (→) when exposed to heat stress at 38°C for 0.5 d. When heat stress continued to 2 d, vacuole degeneration gradually became obvious, and capillary vasodilation (↑) could be seen. Cardiomyocyte necrosis (↓) characterized by nuclear hyperchromatism and karyopyknosis was observed when the heat stress continued to 5 d. The supplement with 0.2 g/L tea polyphenols in drinking water could effectively alleviate the cardiomyocyte damage caused by heat stress, thereby showing no obvious vacuolar degeneration and capillary vasodilation at 2 d and necrosis at 5 d.

### 3.6. Injury-Related Index Analysis in the Chicken Serum

**Figure 7:**
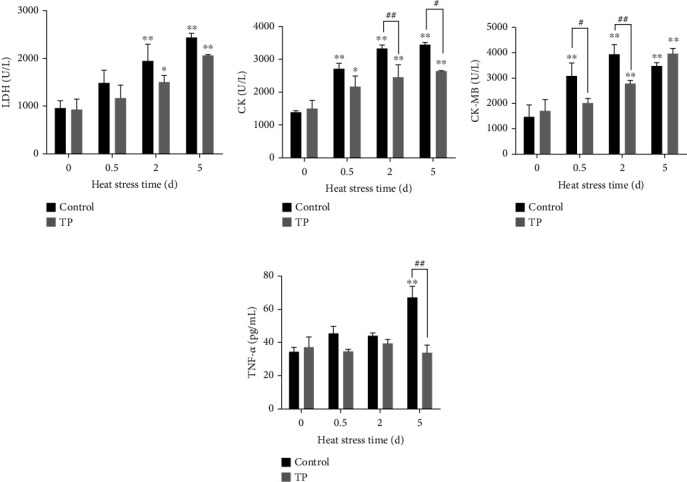
Injury-related index analysis in the serum of chickens. TP means tea polyphenols. Heat stress leads to increases in LDH, CK, CK-MB, and TNF-*α*. The supplement with 0.2 g/L tea polyphenols in drinking water showed a significant downregulation of CK at 2 d (*P* < 0.01) and 5 d (*P* < 0.05), CK-MB at 0.5 d (*P* < 0.05) and 2 d (*P* < 0.01), and TNF-*α* at 5 d (*P* < 0.01). ^∗^*P* < 0.05 and ^∗∗^*P* < 0.01 were compared with 0 h in the control group. #*P* < 0.05 and *^##^P* < 0.01 were compared between the control and tea polyphenol groups under the same conditions.

LDH, CK, CK-MB, and TNF-*α*, which are indexes related to myocardial injury, were detected in the layer hen serum (Figure 7). Heat stress leads to the increases in LDH, CK, CK-MB, and TNF-*α*. Such stress showed significant differences at 2 and 5 d for LDH (*P* < 0.01); at 0.5, 2, and 5 d for CK and CK-MB (*P* < 0.01); and at 5 d for TNF-*α* (*P* < 0.01). The supplement with 0.2 g/L tea polyphenols in drinking water could effectively relieve the upregulation of LDH, CK, CK-MB, and TNF-*α* caused by heat stress. In comparison with the control group under the same conditions, the tea polyphenol group showed a significant downregulation of CK-MB on 0.5 d (*P* < 0.05) and extremely significant downregulation of CK and CK-MB on 2 d (*P* < 0.01). This group also showed a downward trend of CK (*P* < 0.05) and TNF-*α* (*P* < 0.01) at 5 d.

### 3.7. Oxidative Damage-Related Index Analysis in the Chicken Serum

**Figure 8:**
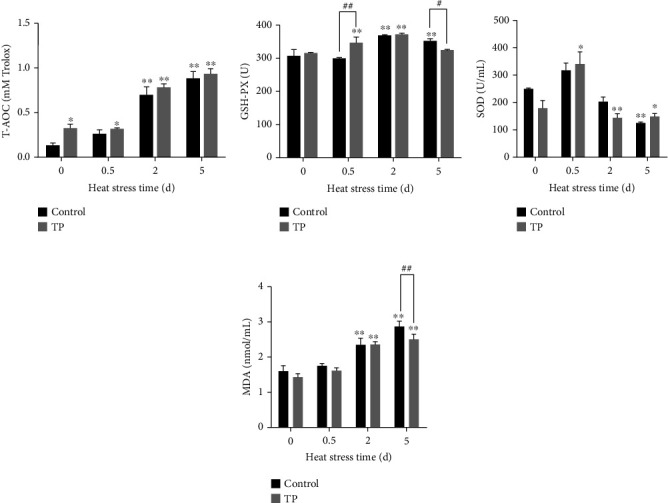
Oxidative damage-related index analysis in the chicken serum. TP means tea polyphenols. Heat stress activated the body's antioxidant level and upregulated the levels of T-AOC, GSH-PX, and MDA. The supplement with 0.2 g/L tea polyphenols in drinking water increased the level of T-AOC under normal condition (*P* < 0.05) and elevated the level of GSH-PX (*P* < 0.01) and SOD at heat stress 0.5 d. However, such supplement decreased the level of MDA at heat stress 5 d (*P* < 0.01). ^∗^*P* < 0.05 and ^∗∗^*P* < 0.01 were compared with 0 h in the control group. ^#^*P* < 0.05 and ^##^*P* < 0.01 were compared between the control and tea polyphenol groups under the same conditions.

Heat stress activated the body's antioxidant level and upregulated the levels of T-AOC, GSH-PX, and SOD (Figure 8). T-AOC and GSH-PX showed a significant upregulation at 2 and 5 d (*P* < 0.01). SOD presented an upward trend at 0.5 d, but it showed a downward trend with the duration of the heat stress and significantly decreased at 5 d (*P* < 0.01). The heat stress upregulated the level of MDA (Figure 8), and its upward trend was dependent on the time of heat stress. Significant differences were observed at 2 d and 5 d (*P* < 0.01). The supplement with 0.2 g/L tea polyphenols in drinking water increased the level of T-AOC in the cardiomyocytes under normal condition (*P* < 0.05) and GSH-PX (*P* < 0.01) and SOD at 0.5 d heat stress. However, such supplement decreased the level of MDA at 5 d heat stress (*P* < 0.01).

### 3.8. Dynamic Expression Levels of Nrf2, Hsps, and Cleaved-Caspase 3 in the Myocardial Tissues

**Figure 9:**
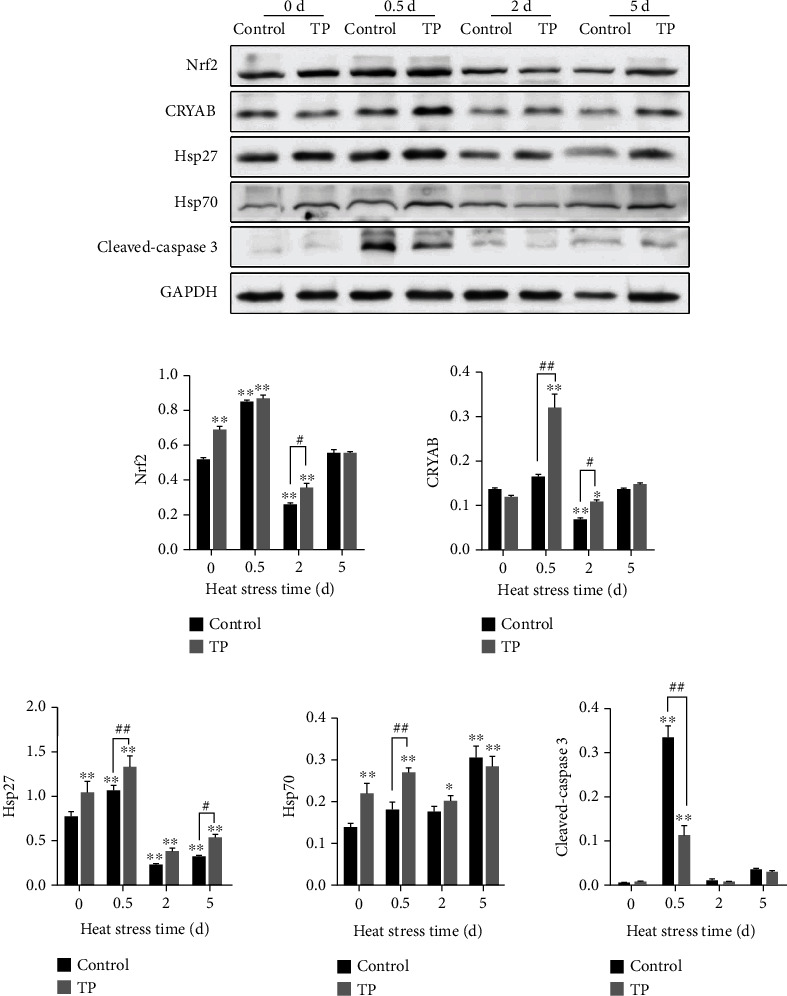
Dynamic expression levels for Nrf2, Hsps, and cleaved-caspase 3 in the myocardial tissues. TP means tea polyphenols. The expression levels were detected using western blot relative to the housekeeping protein GAPDH. The supplement with 0.2 g/L tea polyphenols in drinking water induced Nrf2 (at 0 d with *P* < 0.01 and 2 d with *P* < 0.05) and expressions of CRYAB (at 0.5 and 2 d), Hsp27 (at 0, 0.5, and 5 d), and Hsp70 (at 0 and 0.5 d). ^∗^*P* < 0.05 and ^∗∗^*P* < 0.01 were compared with 0 h in the control group; #*P* < 0.05 and *^##^P* < 0.01 were compared between the control and tea polyphenol groups under the same conditions.

The dynamic expression levels of Nrf2, Hsps, and cleaved-caspase 3 in the myocardial cells fare shown in Figure 9. In the early stage of heat stress, the laying hens activated the body's redox regulation pathway, which was shown by the increase of the Nrf2 level (*P* < 0.01) at 0.5 d. The expression level of Nrf2 showed a downward trend with the continuous increase in the heat stress time, thereby presenting significant difference at 2 d (*P* < 0.01). When the heat stress continued to 5 d, the level of Nrf2 basically recovered. The supplement with 0.2 g/L tea polyphenols in drinking water induced the expression level of Nrf2 under the condition of heat stress for 0 d (*P* < 0.01) and 2 d (*P* < 0.05). Heat stress increased the levels of CRYAB, Hsp27, and Hsp70 at the early stage of 0.5 d and presented a significant increase in Hsp27 (*P* < 0.01). Moreover, the heat stress showed an upward trend but no statistical difference for CRYAB and Hsp70. CRYAB and Hsp27 showed an extremely significant downregulation at 2 d (*P* < 0.01) with the duration of heat stress. The expression of CRYAB, Hsp27, and Hsp70 presented an increase when the heat stress lasted for 5 d compared with 2 d. The supplement with 0.2 g/L tea polyphenols in drinking water induced the expression levels of Hsp27 and Hsp70 under normal condition (heat 0 d, *P* < 0.01) and increased CRYAB, Hsp27, and Hsp70 under heat stress. With regard to the increases under heat stress, significant differences were observed at 0.5 d (*P* < 0.01) and 2 d (*P* < 0.05) for CRYAB, at 0.5 d (*P* < 0.01) and 5 d (*P* < 0.05) for Hsp27, and at 0.5 d (*P* < 0.01) for Hsp70. Heat stress induced the expression of cleaved-caspase 3 and showed an extremely significant increase at 0.5 d (*P* < 0.01). The supplement with 0.2 g/L tea polyphenols in drinking water could effectively inhibit the upregulation of cleaved-caspase 3 caused by heat stress. The inhibition effect was significant at 0.5 d (*P* < 0.01).

## 4. Discussion

The automatic environmental control system and various cooling systems had been promoted and applied in the poultry farm with the development of scientific and standardized breeding. However, the temperature in the house in summer would still be 32–34°C because of the high temperature, thereby subjecting poultry to a subhealth state of heat stress. Researchers had found that heat stress could lead to a decline in the poultry production performance, product quality, and body immunity [1, 2, 23]. This condition could lead to the injury of various tissues and organs. In our study, vacuolar degeneration appeared in cardiomyocytes at 0.5 d of 38°C heat stress. Capillary vasodilation and necrosis occurred at 2 and 5 d, respectively, with the prolongation of heat stress. The results were consistent with the idea that myocardial cells were sensitive to heat, and its damage was an important factor that leads to sudden death [20].

The oxidative stress is defined as the existence of metabolic and radical constituents or so-called reactive (chlorine, oxygen, or nitrogen) species [24, 25]. Heat stress induced excessive ROS, thereby leading to the denaturation and aggregation of proteins and resulting in the imbalances of redox and protein homeostasis. Keap1-Nrf2-ARE and HSF1-HSE are important endogenous protection mechanisms initiated by heat stress [5, 18]. These mechanisms regulate the expression of antioxidant enzymes and Hsps to play a double protective role for cell adaptation and survival. Nrf2 was a key factor in the Keap1-Nrf2-ARE pathway, and its activation of the antioxidant signaling pathways played a key role in preventing oxidative stress-induced cell and tissue damage and maintaining the redox balance. The activation of Hsps and its induced expression were positively correlated with the tolerance to heat stress. Hsps, a molecular chaperone, could help in the protein folding, renaturation, and degradation and stabilize cytoskeletons [22, 26]. CRYAB, Hsp27, and Hsp70 are important members of the Hsps family. Hsp70 plays a vital role in the unfolded or misfolded protein repair and enzyme activity stabilization [6, 27]. Hsps, such as Hsp27 and *α*B-crystallin can maintain the cytoskeletal stability [28, 29]. Our previous study in vivo and in vitro had shown the cytoskeleton protection of CRYAB [22].

In our study, the results verified the activation of Nrf2 in the H9C2 cells when subjected to heat stress at 42°C for 2, 3, and 5 h. Nrf2 demonstrated a significant increase in the myocardial cells when the laying hens were exposed to heat stress at 38°C for 0.5 d. Thereafter, Nrf2 showed a downward trend at 2 d and recovered at 5 d. The heat stress increased the levels of CRYAB, Hsp27, and Hsp70 in the myocardial cells when the laying hens were exposed to heat stress at 38°C for 0.5 d, then had a downregulation at 2 d and had an increase at 5 d. These volatile changes were consistent with the layer's response to heat stress. The laying hens had obvious heat stress symptoms at the beginning of heat stress, such as mouth breathing and increased drinking volume. Accompanied with continuous heat stress to 2 d, the heat stress symptoms have shown relief, which should be related to the body's adaptability. Thus, Nrf2 and Hsps were important targets for reducing damage. *P. japonicus* activated Hsps and Nrf2-regulated HO-1 to alleviate UVB-induced apoptosis [16]. Solanesol could protect the human liver cells from ethanol-induced oxidative injury via upregulation of HO-1 and Hsp70 [17]. Cleaved-caspase 3 was an active fragment produced by shearing during the activation of caspase 3, and its level reflected the level of cell apoptosis. Heat stress could induce cell apoptosis and cleaved-caspase 3. However, it could almost not be detected in H9C2 cells at 2 h of 42°C heat stress, which might be because of the lower apoptosis. This was consistent with the pathological damage that only showed granular degeneration at 2 h of heat stress.

Tea polyphenols, a general term of polyphenols extracted from tea leaves, are a safe and efficient natural antioxidant. Tea polyphenols, a new type of green feed additives, had been used in animal production due to the development of animal nutrition [12, 13, 30]. The supplement with tea polyphenols to the diet could significantly increase the activity of GSH-PX, CAT, and SOD in the serum of broilers. Antioxidant capacity is usually evaluated by determining the activity of antioxidant enzymes [31]. Under a heat stress condition, the supplement with tea polyphenols to broiler diets increased the activity of antioxidant enzymes, such as SOD and GSH in broiler serum, and improved broiler heat stress status. Tea polyphenols are potential drugs against heat stress. In our study, tea polyphenols were used to explore the effect and mechanism of relieving myocardial cell damage caused by heat stress. The results showed that the supplement with tea polyphenols at a concentration of 0.2 g/L in chicken drinking water or 10 *μ*g/mL in a cell culture medium could in vivo and in vitro alleviate the pathological damage and downregulate the levels of LDH, CK, and CK-MB in myocardial cells caused by heat stress. The LDH, CK, and CK-MB in the serum were used as important indicators for measuring myocardial damage because they could be released outside the cells when cardiomyocytes were damaged.

The protection mechanism of tea polyphenols might be related to the upregulation of the antioxidant levels (T-AOC, SOD, and Nrf2) and the induced expression of Hsps (CRYAB, Hsp27, and Hsp70). This notion was consistent with the theory that Nrf2 and Hsps could be activated by natural electrophilic small molecule compounds (or small molecules that could be oxidized to electrophilic compounds) [32]. This fact can also be proven by the research supplement with curcumin to the feed that could activate the expression of Nrf2 and its mediated antioxidant enzyme GSH to enhance the resistance of broilers to heat stress [3, 33, 34]. The supplement with epigallocatechin gallate could effectively increase the total antioxidant capacity of eggs, and its mechanism was related to the upregulation of Nrf2 and HO-1 [12]. In addition, it has been reported in the literature that under heat stress conditions, Nrf2 and HSF1 acted on a common target, such as Hsp70 and HO-1, and there was mutual compensation in cell protection function [35, 36]. However, whether the protective effect of tea polyphenols on alleviating the thermal damage is related to the cross-interaction between Keap1-Nrf2-ARE and HSF1-HSE needs further study.

## 5. Conclusions

In conclusion, the supplement with tea polyphenols at a concentration of 0.2 g/L in the chicken drinking water or at a concentration of 10 *μ*g/mL in the cell culture medium showed a protective effect for cardiomyocytes when they suffered heat stress in vivo and in vitro. The protection mechanism was related to the enhancement of the antioxidant capacity and the induced expression of Hsps.

## Data Availability

The data used to support the findings of this study are available from the corresponding author upon request.
